# A Theoretical Study on the Medicinal Properties and Eletronic Structures of Platinum(IV) Anticancer Agents With Cl Substituents

**DOI:** 10.3389/fonc.2022.860159

**Published:** 2022-05-19

**Authors:** Xiaoyang Yan, Hongwei Gao

**Affiliations:** School of Life Science, Ludong University, Yantai, China

**Keywords:** DFT, IR spectroscopy, geometry optimization, HOMO and LUMO, natural bond orbital

## Abstract

In this paper, we selected Pt(en)Cl_4_, Pt(dach)Cl_4_, and Pt(bipy)Cl_4_ with gradually increasing ligands to explore the ligand effect on the properties of platinum(IV) anticancer drugs. The electronic structures and multiple drug properties of these three complexes were studied at the LSDA/SDD level using the density functional theory (DFT) method. By comparing the gap between the highest occupied molecular orbital (HOMO) and the lowest unoccupied molecular orbital (LUMO), electron affinity, atomic charge population, and natural bond orbital (NBO), we found that the order of reducibility is Pt(bipy)Cl_4_ > Pt(en)Cl_4_ > Pt(dach)Cl_4_. Our research can provide the theoretical basis for the development of anticancer drugs.

## Introduction

Cisplatin is the first generation of the Pt(II) anticancer complex (1). Pt(II) complexes, such as cisplatin, have remarkable curative effects in the treatment of cancer, but the lack of selectivity leads to side effects in the treatment process, such as nephrotoxicity, neurotoxicity, and cross-resistance. These problems severely limit their clinical application (2). In order to overcome the shortcomings of Pt(II) anticancer complexes, scientists have designed and synthesized new platinum complexes with anticancer activity. On the background, Pt(IV) complexes have become a research hotspot (3). Since Pt(IV) anticancer drugs are easily reduced to corresponding Pt(II) drugs, they are called “prodrugs” of Pt(II) anticancer drugs (4). The molecule itself has a low ability to kill cancer cells, but these Pt(IV) complexes are easily reduced to release Pt(II) under physiological conditions; thus, they retain the broad-spectrum and high-efficiency biological activity of their corresponding Pt(II) anticancer complexes and have many unique advantages (5).

The physical and chemical properties of Pt(IV) complexes are significantly different from those of Pt(II) complexes. Compared with the planar Pt(II) complex, the central Pt ion of the Pt(IV) complex has hexacoordinate structures with two axial orbits, which can form a stable octahedral coordination configuration (6). The modification of different ligands at the axial position of Pt(IV) can affect the liposolubility, reduction, targeting, and other biological activities (7–9). This method can provide a unique idea for the design of platinum drugs, which can change the pharmacokinetic and pharmacodynamic properties of drugs. The most representative Pt(IV) complex with anticancer activity is Ormaplatin, also known as Pt(dach)Cl_4_. Pt(dach)Cl_4_ is the first Pt(IV) complex to enter clinical trials and its central Pt atom connects four Cl ligands. Experiments have shown that the Pt(IV) complex is easily reduced when the ligand is Cl (10). Pt(dach)Cl_4_ has successfully undergone six different phase I trials for its good reducibility. However, the subsequent phase II trials of Pt(dach)Cl_4_ had to be interrupted because of its potential neurotoxic adverse effects (11). By studying complexes with similar structures to Pt(dach)Cl_4_, we hope to find complexes with better anticancer properties than Pt(dach)Cl_4_ and provide a theoretical basis for the development of subsequent drugs. Pt(dach)Cl_4_ has played an indispensable role in the development history of Pt(IV) anticancer complexes, but its related theoretical studies are rare. Since Pt(bipy)Cl_4_ and Pt(en)Cl_4_ have similar structures to Pt(dach)Cl_4_, their electronic structures and medical properties were analyzed and compared by calculation software.

The density functional theory (DFT) method has been successfully tested and applied to different systems (12). This method can accurately simulate the physical and chemical important London dispersion interactions (13). A suitable method for calculating the structure of platinum tetravalent compounds has been reported recently (14). In this study, the structures and properties of Pt(dach)Cl_4_, Pt(en)Cl_4_, and Pt(bipy)Cl_4_ were calculated by Gaussian 16 software and Multiwfn 3.8 (15) in the ground state of LSDA with SDD basis though the DFT method. According to the structural parameters obtained by geometric optimization, the charge number of atoms, the HOMO–LUMO gap, and natural bond orbital (NBO) analysis results, we compared the stability, toxicity, electron activity, electron distribution deformation degree, and the force between different orbits of these three complexes. Hiraishi et al. studied far-infrared spectra and force constants of ammine complexes of Pt (IV), Pt (II), and Pd (II) (16).

## Computational Details

In this study, all calculations were performed by Gaussian 16 software (17) and the results were analyzed by the Gaussview 6.0 molecular visualization program (18). Each bond length and bond angle of three anticancer complexes were fully optimized at the local spin-density approximation (LSDA) (19, 20) Stuttgart/Dresden and D95 ECPs (SDD) (21, 22) level of the DFT method. According to the optimized bond length, the reducibilities of the complexes were compared. The distribution of electron density was visually displayed by plotting the electrostatic potential of the optimized complexes. Then, polarizability, electron activity, and electron distribution deformation degree were displayed by comparing the HOMO–LUMO gaps and the softness. In addition, the calculated infrared spectrum data confirm the characteristic vibrational functional group of the complexes.

NBO means that a group of natural orbits are combined into a single electron basis function. This basis function constitutes the electronic configuration of the N particle system; thus, the configuration with less basis than the regular Hartree–Fock orbit can be realized when ci is expanded. Weinhold and Reed further elaborated this basis function and proposed the following concepts: natural spin orbit, natural bond orbit, and natural hybrid orbit (23). They developed these concepts into a set of theories, also called NBO theory. The NBO analysis emphasizes the role of molecular orbital interaction or exchange transfer. It is calculated by considering all possible interactions between filled donor and empty acceptor NBOs. In addition, NBO analysis estimates the energy importance between them *via* the second-order perturbation theory (24). The stabilization energy (Ea) combined with electron delocalization between electron donor NBO (i) and electron acceptor NBO (j) is calculated by following equation (q is the orbital occupancies, E_i_ and E_j_ are diagonal elements, and F_ij_ is the off-diagonal NBO Fock matrix element) (25):


$${\text{E}}^{2}=\left(\Delta {\text{E}}_{\text{ij}}\right)={\text{q}}_{\text{i}}\frac{{\left(\text{F}\left(\text{i},\text{j}\right)\right)}^{2}}{{\text{E}}_{\text{i}}-{\text{E}}_{\text{j}}}={\text{q}}_{\text{i}}=\frac{{\left({\text{F}}_{\text{ij}}\right)}^{2}}{\Delta \text{E}}$$

Multiwfn 3.8 is used to process the optimized complexes for more intuitive illustrations (15). It is a multifunctional program for wavefunction analysis. The main function is to calculate and visualize real space functions: population analysis, bond order analysis, orbital composition analysis, plot density of states, and spectrum and topology analysis for electron density (15). In this paper, Multiwfn 3.8 was used to calculate some parameters: local orbit locator (LOL) (26, 27) and electronic localization function (ELF) (28). Then, the electrostatic potential plane contour maps and the orbital delocalization index (ODI) were calculated by the Mulliken and Hirshfeld methods.

## Results and Discussion

### Optimization and Electronic Structure Analysis

**Figure 1:**
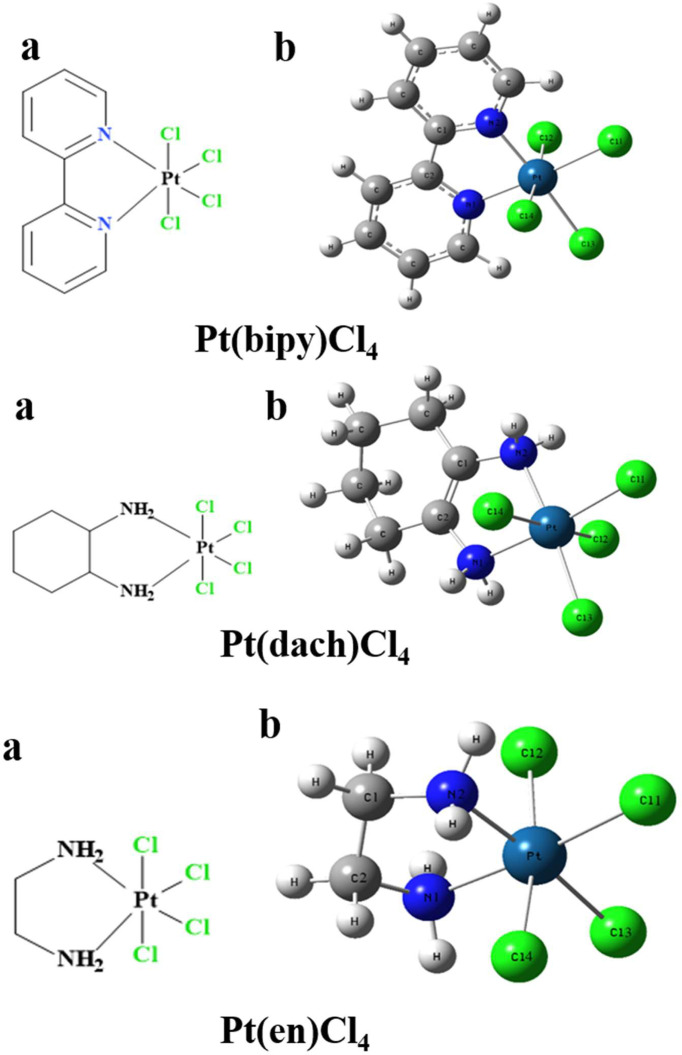
The chemical structure **(A)** and the optimized structure **(B)** of Pt(bipy)Cl_4_, Pt(dach)Cl_4_, and Pt(en)Cl_4_ at the LSDA/SDD level.

The chemical structures of the three complexes (left) and the optimized structures (right) at the LSDA/SDD level are shown in **Figure 1**. The optimized data of bond length and bond angle are shown in **Table 1**. The optimized bond lengths of the Pt-Cl are 2.342 Å (Pt(bipy)Cl_4_), 2.340 Å (Pt(en)Cl_4_), and 2.339 Å (Pt(dach)Cl_4_) in **Table 1**. The Cl-Pt-Cl bond angle (from small to large) is Pt(bipy)Cl_4_ (89.060°) < Pt(dach)Cl_4_ (95.747°) < Pt(en)Cl_4_ (95.980°). Ju Guang Han (1) et al. proved that the longer the bond length of Pt-Cl, the weaker the binding ability between them and Cl, which means that it is easier to break the binding between Pt and Cl in the presence of a DNA/protein environment. Afterwards, Pt(IV) is easier to reduce to Pt (II) to exert anticancer activity. The calculated result shows that the optimized bond lengths of Pt-Cl are 2.342 Å (Pt(bipy)Cl_4_), 2.340 Å (Pt(en)Cl), and 2.339 Å (Pt(dach)Cl_4_). The order is Pt(bipy)Cl_4_ > Pt(en)Cl_4_ > Pt(dach)Cl_4_, which means that Pt(bipy)Cl_4_ is most easily reduced and Pt(dach)Cl_4_ is the most difficult to reduce. The experimental value of R(Pt-Cl1) is 2.33 Å. The deviations are 0.012 Å (Pt(bipy)Cl_4_), 0.009 Å (Pt(dach)Cl_4_), and 0.01 Å (Pt(en)Cl_4_). The experimental value of R(Pt-N1) is 2.17 Å. The deviations are 0.161 Å (Pt(bipy)Cl_4_), 0.13 Å (Pt(dach)Cl_4_), and 0.119 Å (Pt(en)Cl_4_). The bond lengths of Pt-Cl and Pt-N obtained by calculation are close to the experimental values (16). In addition, the intensity of the interaction between Pt and Cl is also a sign that the detachment rate of Cl^-^ from complex in some way in a DNA/protein environment. This is also a method of measuring reaction kinetics that bind Pt(IV) anticancer drugs to DNA (1).

**Table 1:** Optimized bond length (in Å) and bond angle (in °) of complexes Pt(dach)Cl_4_, Pt(bipy)Cl_4_, and Pt(en)Cl_4_ at the LSDA/SDD level.

Geometry	Exp^[16]^	LSDA/SDD		
		Pt(bipy)Cl_4_	Pt(dach)Cl_4_	Pt(en)Cl_4_
R(Pt-Cl1)	2.33	2.342	2.339	2.340
R(Pt-N1)	2.17	2.009	2.040	2.051
A(N1-Pt-N2)	–	89.191°	84.755°	85.679°
A(Cl1-Pt-Cl3)	–	89.060°	95.747°	95.980°
A(Cl1-Pt-N2)	–	94.874°	89.747°	89.133°

Experiments have shown that the reactivity of these three complexes with glutathione (GSH), L-cysteine (L-Cys), and L-methionine (L-Met) follows this rule: Pt(bipy)Cl_4_ > Pt(dach)Cl_4_ > Pt(en)Cl_4_ (29). The order of the Pt-N bond length (from short to long) and the Cl-Pt-Cl bond angle (from small to large) is Pt(bipy)Cl_4_ < Pt(dach)Cl_4_ < Pt(en)Cl_4_, which is just the opposite of the reactivity order proved by experiments (29). However, the order of the bond angles of Cl-Pt-N (from large to small) is Pt(bipy)Cl_4_ > Pt(dach)Cl_4_ > Pt(en)Cl_4_, which is the same as the reactivity order proved by experiments. Thus, we could infer that the bond length of Pt-N from short to long and the bond angle of Cl-Pt-Cl from small to large are negatively correlated with the reactivity. The bond angle of Cl-Pt-N from large to small is positively correlated with the reactivity.

### Vibrational Spectroscopic

**Figure 2:**
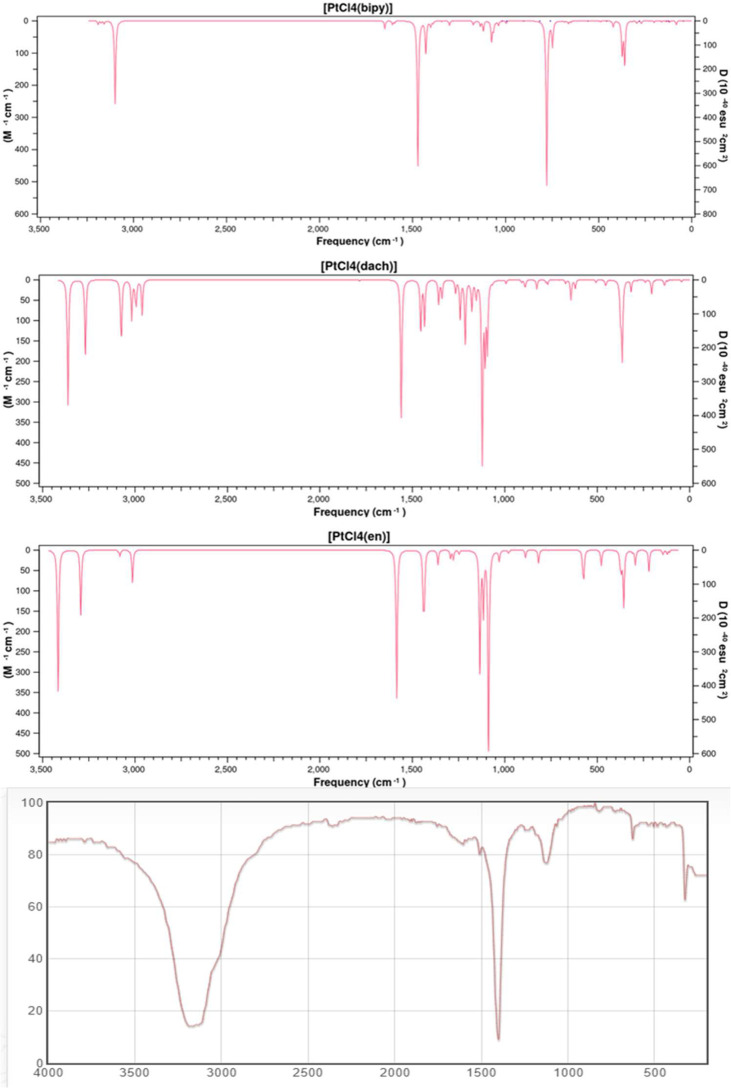
The theoretical frequencies and infrared intensities calculated with the LSDA/SDD method for Pt(bipy)Cl_4_, Pt(dach)Cl_4_, and Pt(en)Cl_4,_ as well as the infrared spectrum of ammonium tetrachloroplatinate.

We calculated the IR frequencies and intensities of Pt(dach)Cl_4_, Pt(bipy)Cl_4_, and Pt(en)Cl_4_ at the LSDA/SDD level. The calculated infrared spectra of these complexes and the experimental infrared spectra of ammonium confirm the characteristic tetrachloroplatinate (Chemical Database of Chinese Academy of Sciences) are depicted in **Figure 2**. The calculated and experimental data are shown in **Table 2**. The vibration types exhibited by the highest peaks of these three complexes are the out-of-plane bending vibrations of C-H and N-H: γ(CH) and γ(NH) are at 779 cm^-1^ in Pt(bipy)Cl_4_, γ(CH) and γ(NH) are at 1,122 cm^-1^ in Pt(dach)Cl_4_, and γ(CH) and γ(NH) are at 1,088 cm^-1^ in Pt(en)Cl_4_, and Pt(en)Cl_4_ has the highest infrared intensity. By comparing Pt(dach)Cl_4_ and Pt(en)Cl_4_, we can clearly find that the highest peak of Pt(bipy)Cl_4_ shifts to the low-frequency region. Moreover, CH-related vibration types include in-plane bending vibration [δ(CH)] and symmetric stretching vibration [υ(CH)]. The position of δ(CH) is at 1,471 cm^-1^ in Pt(bipy)Cl_4_, 1,560 cm^-1^ in Pt(dach)Cl_4_, and 1,585 cm^-1^ in Pt(en)Cl_4_, and Pt(bipy)Cl_4_ has the highest infrared spectral intensity and shifts to low frequency. The position of υ(CH) is at 3,098 cm^-1^ in Pt(bipy)Cl_4_, 3,363 cm^-1^ in Pt(dach)Cl_4_, and 3,416 cm^-1^ in Pt(en)Cl_4_, and Pt(en)Cl_4_ has the highest infrared spectral intensity. In addition, the absorption peak of Pt(bipy)Cl_4_ shifts obviously to the low-frequency region compared with Pt(en)Cl_4_.

**Table 2:** Frequencies (cm^-1^) and infrared intensities^a^ (km/mol) calculated for complexes Pt(dach)Cl_4_, Pt(bipy)Cl_4_, and Pt(en)Cl_4_ at the LSDA/SDD level.

Assignment^b^	Exp	Pt(bipy)Cl_4_	Pt(dach)Cl_4_	Pt(en)Cl_4_
γ(CH), γ(NH)		779 (148)	1,122 (128)	1,088 (139)
δ(CH)	1401	1,471 (130)	1,560 (95)	1,585 (105)
υ(CH)	3163	3,098 (68)	3,363 (89)	3,416 (97)
υ_sym_(Cl-Pt-Cl)	325	374 (21)	375 (18)	376 (9)
υ_asym_(Cl-Pt-Cl)		360 (37)	365 (54)	357 (39)
υ_asym_(CH)		3,097 (7)	3,018 (16)	
γ(Cl-Pt-Cl)		122 (0.99)	135 (2.72)	145 (3.10)
scr(Cl-Pt-Cl)		162 (0.42)	94 (0.17)	112 (0.02)
p(NH)			187 (0.16)	310 (1.34)

a IR intensity, the value in parentheses.

b Vibrational modes, ν = stretch; scr = scissoring; γ = out-of-plane angle bending, δ = in-plane deformation, sym = symmetric and asym = asymmetric, p = in-plane rocking vibration, ω = out-of-plane rocking vibration. Out-of-phase and in-phase notations refer to the phase between dissimilar vibrations or analogous vibrations when more than one of the same moieties exist.

We observe that the vibration types related to Pt are symmetric stretching vibration [υ_sym_(Cl-Pt-Cl)], antisymmetric stretching vibration [υ_asym_(Cl-Pt-Cl)], scissoring vibration [scr(Cl-Pt-Cl)], and out-of-plane bending vibration [γ(Cl-Pt-Cl)]. The position of υ_sym_(Cl-Pt-Cl) is at 374 cm^-1^ in Pt(bipy)Cl_4_, 375 cm^-1^ in Pt(dach)Cl_4_, and 376 cm^-1^ in Pt(en)Cl_4_, and Pt(bipy)Cl_4_ has the highest infrared intensity. The position of υ_asym_(Cl-Pt-Cl) is at 360 cm^-1^ in Pt(bipy)Cl_4_, 365 cm^-1^ in Pt(dach)Cl_4_, and 357 cm^-1^ in Pt(en)Cl_4_, and Pt(dach)Cl_4_ has the highest infrared intensity. The position of γ(Cl-Pt-Cl) is at 122 cm^-1^ in Pt(bipy)Cl_4_, 135 cm^-1^ in Pt(dach)Cl_4_, and 145 cm^-1^ in Pt(en)Cl_4_, and Pt(en)Cl_4_ has the highest infrared intensity. In addition, compared with Pt(en)Cl_4_, the absorption peak of Pt(bipy)Cl_4_ shifts slightly to the low-frequency region. The position of scr(Cl-Pt-Cl) is at 162 cm^-1^ in Pt(bipy)Cl_4_, 94 cm^-1^ in Pt(dach)Cl_4_, and 112 cm^-1^ in Pt(en)Cl_4_, and Pt(bipy)Cl_4_ has the highest infrared intensity. In addition, compared with Pt(bipy)Cl_4_, the absorption peak of Pt(dach)Cl_4_ shifts obviously to the low-frequency region.

The infrared spectrum of ammonium tetrachloroplatinate has three peaks between 300 cm^-1^ and 3,500 cm^-1^, and the vibration modes are in-plane bending vibration [δ(CH)], symmetric stretching vibration [υ(CH)], and symmetric stretching vibration [υ_sym_(Cl-Pt-Cl)]. Ammonium tetrachloroplatinate, Pt(bipy)Cl_4_, Pt(dach)Cl_4_, and Pt(en)Cl_4_ have similar structures. The positions of δ(CH), υ_sym_(Cl-Pt-Cl), and υ(CH) are 1,401 cm^-1^, 3,163 cm^-1^, and 325 cm^-1^, respectively, in ammonium tetrachloroplatinate. Compared with the experimental results, the smaller the substituent attached to Pt, the more the positions of δ(CH) and υ_sym_(Cl-Pt-Cl) move to the high-frequency region. The size of the substituent does not affect the position of υ(CH).

### HOMO–LUMO Calculations

**Figure 3:**
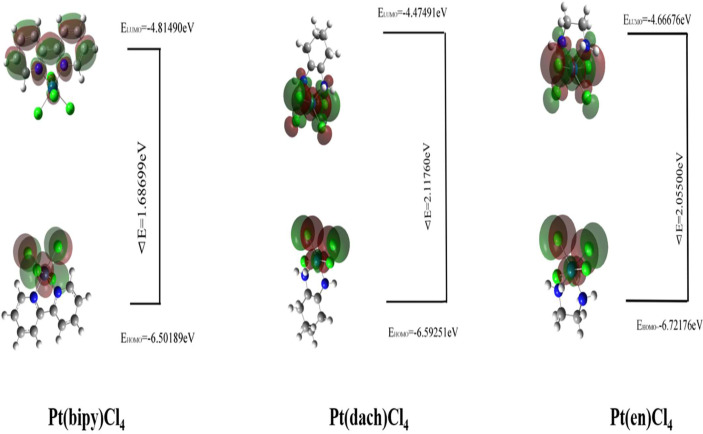
The HOMO and LUMO of Pt(bipy)Cl_4_, Pt(dach)Cl_4_, and Pt(en)Cl_4_.

The HOMO–LUMO gap values play a vital role in a wide range of chemical interactions (30). The size of HOMO and LUMO shows the ability to lose and gain electrons. The energy gap is an important stability index for the individual species concerned (31, 32). Generally speaking, a large HOMO–LUMO gap indicates high stability. High stability of a molecule reflects low reducibility toward chemical reactions in some sense. A small HOMO–LUMO gap indicates that the overall energy difference between the occupied and non-occupied orbits is smaller. The smaller overall energy difference lends smaller excitation energy and larger polarizability (33). The HOMO–LUMO gap values are calculated at the LSDA/SDD level. The different physical parameters calculated from HOMO–LUMO are displayed in **Table 3**. The HOMO and LUMO of three complexes are displayed in **Figure 3**.

**Table 3:** HOMO–LUMO energy gaps and other physical relationships of the Pt(dach)Cl_4_, Pt(bipy)Cl_4_, and Pt(en)Cl_4_ at the LSDA/SDD level.

Molecular properties	Pt(bipy)Cl_4_	Pt(dach)Cl_4_	Pt(en)Cl_4_	Formula
E_HOMO_ (eV)	−6.50189	−6.59251	−6.72176	
E_LOMO_ (eV)	−4.81490	−4.47491	−4.66676	
Energy gap (eV)	1.68699	2.11760	2.05500	
Ionization potential (IP)	6.50189	6.59251	6.72176	IP = −E_HOMO_
Electron affinity	4.81490	4.47491	4.66676	Ea = −E_LUMO_
Electro negativity	5.65840	5.53371	5.69426	X = $\frac{\text{IP}+\text{Ea}}{2}$ ′
Hardness	0.84350	0.92171	1.0275	η = $\frac{\text{IP}+\text{Ea}}{2}$
Chemical potential	−5.65840	−5.53371	−5.69426	μ = −(X)
Softness	1.18554	1.08494	0.97324	S = $\frac{1}{\text{η}}$

The HOMO–LUMO gap of Pt(bipy)Cl_4_, Pt(en)Cl_4_, and Pt(dach)Cl_4_ are 1.68699 eV, 2.05500 eV, and 2.11760 eV, respectively. Thus, the order of polarizability from large to small is Pt(bipy)Cl_4_ > Pt(en)Cl_4_ > Pt(dach)Cl_4_, the order of stability from large to small is Pt(dach)Cl_4_ > Pt(en)Cl_4_ > Pt(bipy)Cl_4_, and the order of reducibility from large to small is Pt(bipy)Cl_4_ > Pt(en)Cl_4_ > Pt(dach)Cl_4_. Ju Guang Han (1) et al. proved that low electron affinity leads to poor ability to obtain electrons; thus, the stability is strong. The electron affinity of Pt(bipy)Cl_4_, Pt(en)Cl_4_, and Pt(dach)Cl_4_ are 4.81490 kcal mol^-1^, 4.66676 kcal mol^-1^, and 4.47491 kcal mol^-1^, respectively; hence, order of stability from large to small is Pt(dach)Cl_4_ > Pt(en)Cl_4_ > Pt(bipy)Cl_4_. Therefore, the rules of the HOMO–LUMO gap and electron affinity proved that Pt(bipy)Cl_4_ has the largest reducibility and Pt(dach)Cl_4_ has the smallest reducibility. The order of softness from large to small is Pt(bipy)Cl_4_ (1.18554) > Pt(dach)Cl_4_ (1.08494) > Pt(en)Cl_4_ (0.97324). Softness reflects the activity of electrons and the degree of deformation of their distribution. Generally speaking, the larger the softness indicates, the easier it is for a molecule to react. Thus, softness also shows that Pt(bipy)Cl_4_ is more responsive than Pt(dach)Cl_4_.

### Natural Charge Analysis

Mulliken atomic charge calculation is a vital function in the application of quantum chemical calculation, because it affects physical properties such as electronic structures, molecular polarizability, and many other properties of a molecular system (34, 35). Natural charge analysis is an alternative to the traditional Mulliken population analysis. It shows better numerical stability and better description of the electron distribution in complexes with high ionic properties, such as complexes containing metal atoms (36).

**Figure 4:**
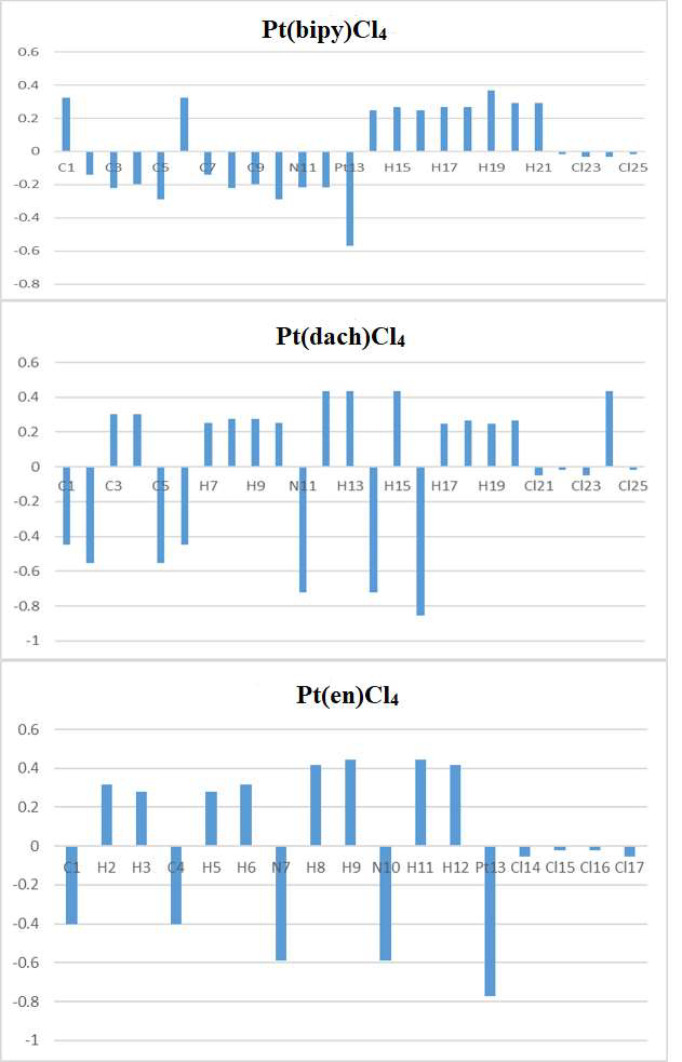
Natural atomic charges for Pt(bipy)Cl_4_, Pt(dach)Cl_4_, and Pt(en)Cl_4_.

The calculated natural charges of Pt(bipy)Cl_4_, Pt(dach)Cl_4_, and Pt(en)Cl_4_ at the LSDA/SDD level are displayed in **Tables 4**–**6**. Natural charge distribution histograms are shown in **Figure 4**. Among the three complexes, Cl and N connected to Pt are negatively charged. In the four Cl substituents of Pt(bipy)Cl_4_, Cl22 and Cl25 have a high amount of charge (−0.017 a.u.) while the lowest charge is seen on Cl23 (−0.034 a.u.). In the four Cl substituents of Pt(dach)Cl_4_, Cl22 and Cl25 have a high amount of charge (−0.016 a.u.) while the lowest charge is seen on Cl23 and Cl21 (−0.049 a.u.). In the four Cl substituents of Pt(en)Cl_4_, Cl15 and Cl16 have a high amount of charge (−0.020 a.u.) while the lowest charge is seen on Cl14 and Cl17 (−0.055 a.u.).

**Table 4:** Natural atomic charges for Pt(bipy)Cl_4_.

Natural atomic charge (a.u.)			
Atom	Charge	Atom	Charge
C1	0.325	H14	0.247
C2	−0.139	H15	0.267
C3	−0.221	H16	0.247
C4	−0.195	H17	0.267
C5	−0.290	H18	0.267
C6	0.325	H19	0.367
C7	−0.139	H20	0.293
C8	−0.221	H21	0.293
C9	−0.195	Cl22	−0.017
C10	−0.290	Cl23	−0.034
N11	−0.217	Cl24	−0.033
N12	−0.217	Cl25	−0.017
Pt13	−0.569		

**Table 5:** Natural atomic charges for Pt(dach)Cl_4_.

Natural atomic charge (a.u.)			
Atom	Charge	Atom	Charge
C1	−0.447	N14	−0.723
C2	−0.551	H15	0.434
C3	0.302	Pt16	−0.856
C4	0.302	H17	0.250
C5	−0.551	H18	0.265
C6	−0.447	H19	0.250
H7	0.251	H20	0.265
H8	0.275	Cl21	−0.049
H9	0.275	Cl22	−0.016
H10	0.251	Cl23	−0.049
N11	−0.723	H24	0.437
H12	0.434	Cl25	−0.016
H13	0.437		

**Table 6:** Natural atomic charges for Pt(en)Cl_4_.

Natural atomic charge (a.u.)			
Atom	Charge	Atom	Charge
C1	−0.402	Cl14	−0.055
H2	0.315	Cl15	−0.020
H3	0.279	Cl16	−0.020
C4	−0.402	Cl17	−0.055
H5	0.279		
H6	0.315		
N7	−0.591		
H8	0.418		
H9	0.444		
N10	−0.591		
H11	0.444		
H12	0.418		
Pt13	−0.772		

Ju Guang Han et al. proved that the more charge Pt carries, the greater the activity (1). The dose required to inhibit tumor is inversely related to Pt activity. Pt(bipy)Cl_4_ carries the most charge (−0.569 a.u.), so it has the highest activity and the minimum dose required for the treatment of tumors. Pt(dach)Cl_4_ carries the least charge (−0.772 a.u.), so it has the lowest activity and the largest dose required for tumor treatment. The cytotoxicity carried by anticancer Pt complexes is related to the Cl atomic charge. The smaller the negative charge is, the smaller the toxicity is (1). Pt(bipy)Cl_4_ is the least toxic (−0.101 a.u.) and Pt(dach)Cl_4_ is more toxic (−0.13 a.u.) than Pt(bipy)Cl_4_. Pt(bipy)Cl_4_ is better than Pt(dach)Cl_4_ in terms of toxicity and dose of the drug needed to treat cancer; thus, we infer that Pt(bipy)Cl_4_ has a greater potential in the treatment of cancer.

### NBO Analysis

NBO analysis provides an effective method for studying intramolecular bonds, intermolecular bonds, and bond–bond interactions. It also provides a convenient basis for studying charge transfer or conjugate interactions in molecular systems (37). The second-order Fork matrix is used to evaluate the donor–acceptor interactions in NBO analysis (38). The interactions result in a loss of occupancy from the localized NBO of the idealized Lewis structure into an empty non-Lewis orbital. (39) The electron donor orbit (i), electron acceptor orbit (j), and the corresponding second-order stabilization energy distribution [E^(2)^] in the stable configurations of the three complexes are shown in **Tables 7**–**9**. The E^(2)^ value represents the degree (strong or weak) of electron delocalization and the interaction between electron donor and electron acceptor, which show the contribution tendency of the electrons transferring from electron donor to the electron acceptor (40).

**Table 7:** Second-order perturbation theory analysis of Fock matrix in NBO basis for Pt(bipy)Cl_4_ at the LSDA/SDD level.

Donor(i)	Acceptor(j)	^a^E^(2)^ (kcal/mol)	^b^E(i) − E(j)(a.u)	^c^F(i.j)(a.u)
BD (1)C1-C5	BD*(1)C4-H15	3.19	1.08	0.053
BD (1)C1-C5	BD*(1)C6-N12	2.68	1.01	0.046
BD (1)C1-C5	RY*(10)Pt13	1.67	13.49	0.135
BD (1)C1-C5	RY*(21)Pt13	1.59	21.79	0.167
BD (1)C1-N11	BD*(1)N11-Pt13	4.91	1.41	0.080
BD (1)C2-C3	BD*(2)C4-C5	10.06	0.22	0.043
BD (1)C2-H20	BD*(1)C1-N11	5.41	0.89	0.062
BD (1)C6-N12	BD*(1)Pt13-Cl22	0.98	1.00	0.029
BD (2)C6-N12	BD*(2)C7-C8	9.38	0.30	0.048
BD(1)N11-Pt13	RY*(10)Pt13	36.25	13.38	0.645
BD(1)N12-Pt13	RY*(21)Pt13	35.85	21.67	0.817
BD(1)Pt13-Cl22	BD*(1)N12-Pt13	43.68	1.04	0.195
CR(1)Pt13	BD*(1)C6-N12	2.01	4.16	0.082
LP(3)Cl22	BD*(1)Pt13-Cl22	8.83	0.97	0.085
BD*(1)N11-Pt13	RY*(2)C5	131.84	0.18	0.368
BD*(1)N11-Pt13	RY*(14)Pt13	477.31	0.03	0.291
BD*(1)N11-Pt13	BD*(1)N12-Pt13	6,875.43	0.01	0.493
BD*(1)Pt13-Cl25	BD*(1)Pt13-Cl23	17.15	0.09	0.079

a E^(2)^, energy of hyper conjugative interaction (stabilization energy).

b E(j) − E(i), energy difference between donor and acceptor i and j NBO orbitals.

c F(i,j) is the Fock matrix element between i and j NBO orbitals.

**Table 8:** Second-order perturbation theory analysis of Fock matrix in NBO basis for Pt(dach)Cl_4_ at the LSDA/SDD level.

Donor(i)	Acceptor(j)	^a^E^(2)^ (kcal/mol)	^b^E(i) − E(j)(a.u)	^c^F(i.j) (a.u)
BD (1)C1-C2	LP(2)C6	13.44	0.40	0.090
BD (1)C1-H19	LP(2)C6	12.07	0.34	0.079
BD (1)C3-C4	BD*(1)Pt16-Cl21	1.00	0.83	0.029
BD (1)C4-N11	LP*(6)Pt16	8.92	0.94	0.091
BD (1)C6-H10	LP(2)C6	62.04	0.12	0.098
LP(1)C6	BD*(1)C5-C6	66.98	0.28	0.141
LP*(4)Pt16	BD*(1)Pt16-Cl21	29.82	0.11	0.078
LP*(5)Pt16	BD*(1)N11-Pt16	221.70	0.03	0.097
LP*(5)Pt16	BD*(1)N14-Pt16	1,469.24	0.03	0.271
LP*(6)Pt16	BD*(1)N11-Pt16	2,002.80	0.02	0.278
BD*(1)C5-C6	BD*(1)C6-C10	104.35	0.16	0.216
BD*(1)N11-Pt16	RY*(3)C3	60.81	0.85	0.384
BD*(1)N11-Pt16	RY*(18)Pt16	93.44	12.21	1.808
BD*(1)Pt16-Cl21	BD*(1)N14-Pt16	2,135.53	0.03	0.341
LP* (1)C6	LP*(1)H17	925.48	0.24	0.445
LP* (1)H17	BD*(1)Pt16-Cl21	0.19	0.13	0.006
LP* (1)H17	BD*(1)N11-Pt16	0.32	0.16	0.008
LP(4)Cl25	LP*(6)Pt16	88.20	0.48	0.202

a E^(2)^ = energy of hyper conjugative interaction (stabilization energy).

b E(j) − E(i) = energy difference between donor and acceptor i and j NBO orbitals.

c F(i,j) is the Fock matrix element between i and j NBO orbitals.

**Table 9:** Second-order perturbation theory analysis of Fock matrix in NBO basis for Pt(en)Cl_4_ at the LSDA/SDD level.

Donor(i)	Acceptor(j)	^a^E^(2)^ (kcal/mol)	^b^E(i) − E(j)(a.u)	^c^F(i.j)(a.u)
BD (1)C1-H2	BD*(1)N7-H8	25.99	0.87	0.135
BD (1)C1-H3	BD*(1)N10-Pt13	1.54	0.80	0.036
BD (1)C4-H5	BD*(1)C4-H6	11.64	0.74	0.083
BD (1)C4-H6	BD*(1)C4-H5	9.46	0.75	0.078
BD (1)N7-H8	LP*(4)Pt13	76.50	1.18	0.281
LP*(4)Pt13	RY*(1)C4	173.32	0.10	0.294
LP(2)Cl17	BD*(1)Pt13-Cl14	3.70	0.91	0.054
BD*(1)N10-Pt13	LP*(4)Pt13	295.55	0.22	0.363
BD*(1)Pt13-Cl14	BD*(1)Pt13-Cl15	224.12	0.04	0.173
BD*(1)Pt13-Cl15	BD*(1)N10-Pt13	709.29	0.06	0.286
BD*(1)Pt13-Cl15	BD*(1)Pt13-Cl17	584.76	0.01	0.150
BD*(1)Pt13-Cl16	BD*(1)Pt13-Cl17	172.64	0.06	0.174
BD*(1)Pt13-Cl17	LP*(4)Pt13	22.90	0.26	0.129
BD*(1)Pt13-Cl17	RY*(1)N7	16.32	2.22	0.463
BD*(1)Pt13-Cl17	RY*(1)N10	19.32	2.25	0.504
BD*(1)Pt13-Cl17	BD*(1)N10-Pt13	912.87	0.04	0.289
BD*(1)N10-Pt13	RY*(20)Pt13	66.23	24.16	2.305
BD*(1)Pt13-Cl15	LP*(4)Pt13	17.63	0.28	0.114

a E^(2)^ = energy of hyper conjugative interaction (stabilization energy).

b E(j) − E(i) = energy difference between donor and acceptor i and j NBO orbitals.

c F(i,j) is the Fock matrix element between i and j NBO orbitals.

The largest interaction energies of the three complexes appear between the anti-bonding donor and the anti-bonding acceptor, showing great delocalization effects. The largest interaction energy is the anti-bonding N11-Pt13 and anti-bonding N12-Pt13 in the Pt(bipy)Cl_4_ complex (6,875.43 kcal/mol). This result clearly demonstrates strong delocalization between BD*(1)N11-Pt13 and BD*(1)N12-Pt13, and also shows a large tendency of anti-bonding N11-Pt13 to provide electrons to anti-bonding N12-Pt13. The interactions between the central atom Pt and the surrounding atoms are BD*(1)Pt16-Cl21 to BD*(1)N14-Pt16 and BD*(1)Pt13-Cl17 to BD*(1)N10-Pt13, and their energies are 2,135.53 kcal/mol and 912.87 kcal/mol, respectively, in the Pt(dach)Cl_4_ and Pt(dach)Cl_4_ complex. According to the comparison of calculated data, the degree of electron delocalization between the anti-bonding donor and the anti-bonding acceptor of Pt(bipy)Cl_4_ is higher than that of Pt(dach)Cl_4_ and Pt(en)Cl_4_.

### Quantifying the Extent of Spatial Delocalization of Orbitals *via* the Orbital Delocalization Index

The ODI of an orbit is calculated by this formula (41):


$$\text{ODI}=0.01\times {\sum_{A}\left(\Theta_{A,i}\right)}^{2}$$

Where *Θ*_*A*,*i*_ ′ is the composition of atom *A* in orbital *i*.

**Figure 5:**
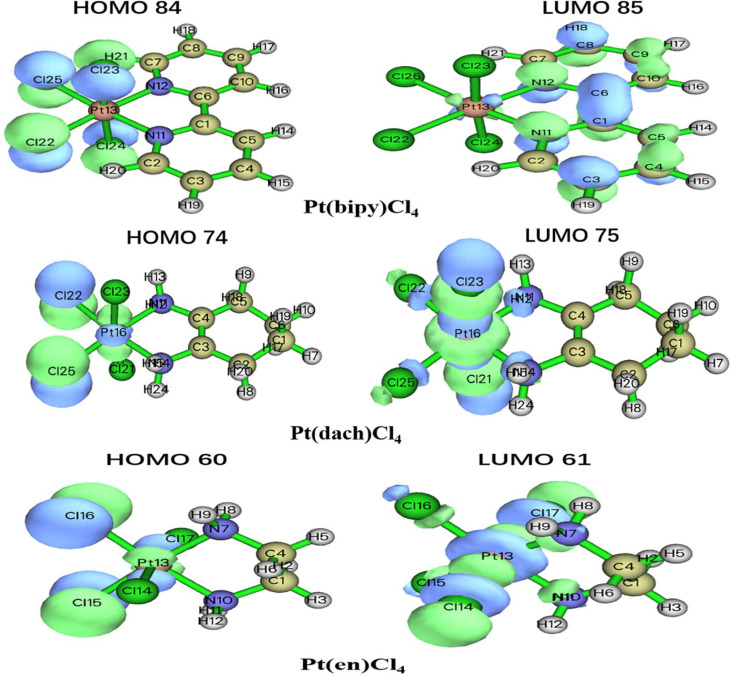
Orbital isosurface map of partial orbitals of Pt(bipy)Cl_4_, Pt(dach)Cl_4_, and Pt(en)Cl_4_.

The delocalization degree of orbit means that the range of orbit distribution is narrow or wide. The larger the distribution range, the stronger the delocalization (42). The ODI is a useful indicator of quantifying the extent of orbital spatial delocalization. The lower (higher) the ODI is, the stronger the orbital delocalization (localization) is. For example, if an orbit is completely localized on an atom, the orbital component of that atom is 100%. Then, the ODI value is (100^2^)/100 = 100, which reaches the theoretical maximum. If this orbit is evenly distributed on two atoms, the ODI value will be (50^2^ + 50^2^)/100 = 50, which is obviously smaller. Strong delocalization indicates that orbits occupy more atoms; thus, the ODI value will be small (41). We calculate the ODI of Pt(dach)Cl_4_, Pt(bipy)Cl_4_, and Pt(en)Cl_4_ through the Mulliken and Hirshfeld methods at the LSDA/SDD level. The calculation process was completed using the Multiwfn 3.8 software, and the obtained data are shown in **Table 10**. **Figure 5** shows the orbital isosurface map of the partial orbitals of three complexes.

**Table 10:** Orbital delocalization index of Pt(dach)Cl_4_, Pt(bipy)Cl_4_, and Pt(en)Cl_4_ through the Mulliken method and the Hirshfeld method.

Compound	Type	Mulliken	Hirshfeld
Pt(bipy)Cl_4_	A	24.91	23.72
	B	10.28	8.01
Pt(dach)Cl_4_	A	39.69	38.19
	B	25.85	26.81
Pt(en)Cl_4_	A	39.41	37.97
	B	25.92	26.78

A, MO 84 in Pt(bipy)Cl_4_, MO 74 in Pt(dach)Cl_4_, and MO 60 in Pt(en)Cl_4_.

B, MO 85 in Pt(bipy)Cl_4_, MO 75 in Pt(dach)Cl_4_, and MO 61 in Pt(en)Cl_4_.

By comparing the ODI values and orbital isosurface maps, it can be seen that the ODI value is indeed able to quantify the extent of orbital delocalization faithfully. HOMO 84, HOMO 74, and HOMO 60 show a partial delocalization character. Four Cl ions connected by the central Pt atom of each complex mainly and equally contribute to the orbital; therefore, their ODI is not quite high (41). Comparing the data obtained using the Mulliken method, the ODI of Pt(bipy)Cl_4_ is smallest in the three complexes (HOMO 84 = 24.91, LUMO 85 = 10.28). Comparing the data obtained using the Hirshfeld method, the ODI of Pt(bipy)Cl_4_ is smallest in the three complexes (HOMO 84 = 23.72, LUMO 85 = 8.01). Therefore, the HOMO and LUMO orbitals of Pt(bipy)Cl_4_ have strong delocalization.

### Molecular Surface Electrostatic Potential Analysis

Molecular electrostatic potential is defined as the energy needed to transfer positive charges from an infinite distance to a point in the space around the molecule. Bonaccorsi et al. (43) first defined the electrostatic potential energy of molecules:


$$\text{V}\left(\text{r}\right)=\sum^{}\text{A}\frac{{\text{Z}}_{\text{A}}}{\left|{\text{R}}_{\text{A}}-\text{r}\right|}-\int^{}\frac{\text{ρ}\left({\text{r}}^{\prime }\text{d}{\text{r}}^{\prime }\right)}{\left|{\text{r}}^{\prime }-\text{r}\right|}\left(1.25\right)$$

where Z and R represent the charge and position of the nucleus, respectively, and r represents the distance of a unit positive charge from a molecule that produces an electrostatic field (44). The molecular electrostatic potential is widely used to predict molecular nucleophilic sites, nucleophilic sites, and molecular recognition modes.

**Figure 6:**
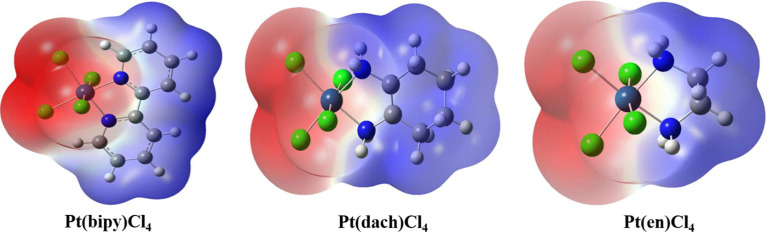
Three-dimensional electrostatic potential diagram of Pt(bipy)Cl_4_, Pt(dach)Cl_4_, and Pt(en)Cl_4_.

The molecular surface electrostatic potential can be expressed by a three-dimensional electrostatic potential diagram. The three-dimensional electrostatic potential diagram of the three complexes at the LSDA/SDD level is shown in **Figure 6**. The molecular surface positive electrostatic potential is shown in red, while the negative electrostatic potential is shown in blue. As shown in **Figure 6**, the Pt-Cl bond is mainly surrounded by the negative electrostatic potential, which means that this region has the ability to contribute electrons and nucleophilic properties. The C-H bond is mainly surrounded by the positive electrostatic potential; thus, this region has the ability to obtain electrons and electrophilic properties. Methods other than electrostatic potential cannot visually display the amphiphilicity of halogen-containing systems.

**Figure 7:**
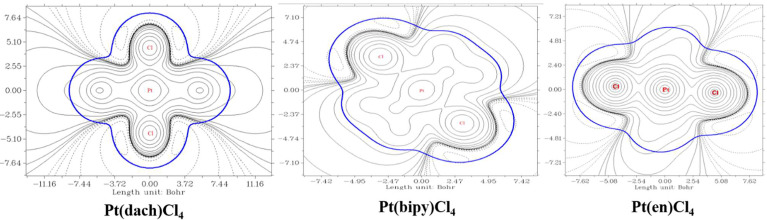
Electrostatic potential contour maps of Pt(bipy)Cl_4_, Pt(dach)Cl_4_, and Pt(en)Cl_4_.

We used Multiwfn 3.8 to draw electrostatic potential contour maps of the three complexes in **Figure 7**. The black solid line and the dashed line in this figure represent the regions where the electrostatic potential is positive and negative, respectively. The bold blue line corresponds to the vdW surface (isosurface of electron density = 0.001 a.u., as defined by R. F. W. Bader). From the graph, it is clear that the Pt atom is overall positively charged, because the vdW surface close to the Pt atom largely intersects solid contour lines. For the same reason, we can see that Pt(en)Cl_4_ has more electrons in Pt and Cl atoms than Pt(bipy)Cl_4_ and Pt(dach)Cl_4_.

Extreme point information of the molecular surface electrostatic potential (45) of the three complexes are shown in **Tables 11**–**13**. The surface electrostatic potential of Pt(bipy)Cl_4_ has 2 minimum points (minimum = −2.214701 eV) and 11 maximum points (maximum = 2.434223 eV); Pt(dach)Cl_4_ has 5 minimum points (minimum = −2.140967 eV) and 9 maximum points (maximum = 2.275112 eV); Pt(en)Cl_4_ has 3 minimum points (minimum = −2.029787 eV) and 8 maximum points (maximum = 2.373782 eV).

**Table 11:** Extreme point information of the molecular surface electrostatic potential of Pt(bipy)Cl_4_.

The number of surface minima				Coordinate (Å)		
NO	a.u.	eV	kcal/mol	*X*	*Y*	*Z*
1*	−0.08138876	−2.214701	−51.072258	−3.655365	0.004156	−0.995303
2	−0.08138040	−2.214473	−51.067015	−3.646132	0.014582	1.008254
The number of surface maxima						
1	−0.02801387	−0.762296	−17.578985	0.025790	−0.020110	4.300648
2	−0.02800087	−0.761942	−17.570827	0.065575	0.008607	−4.288198
3	0.03068932	0.835099	19.257853	1.901131	−2.114969	−1.670970
4	0.03076798	0.837239	19.307218	1.923995	−2.101659	1.671094
5	0.03066312	0.834386	19.241417	1.900052	1.985469	−1.672835
6	0.03079556	0.837990	19.324523	1.925578	2.000638	1.673175
7	0.06258652	1.703066	39.273669	1.804210	5.830401	−0.042303
8	0.06254430	1.701917	39.247174	1.915195	−5.812078	−0.060004
9	0.07082685	1.927297	44.444559	5.113171	−4.078162	−0.055893
10*	0.08945605	2.434223	56.134566	5.210850	0.005169	−0.067639
11	0.07081336	1.926930	44.436092	5.137576	4.024138	−0.036262

*denotes that two extreme points are minimum and maximum, respectively.

**Table 12:** Extreme point information of the molecular surface electrostatic potential of Pt(dach)Cl_4_.

The number of surface minima				Coordinate (Å)		
NO	a.u.	eV	kcal/mol	*X*	*Y*	*Z*
1*	−0.07867908	−2.140967	−49.371911	−3.861251	0.041053	−0.075078
2	0.04478665	1.218707	28.104068	4.404145	2.229902	2.113401
3	0.04480653	1.219248	28.116548	4.465099	−2.207334	−2.059333
4	0.03488121	0.949166	21.888308	5.853910	−2.075173	0.559527
5	0.03488734	0.949333	21.892157	5.888562	2.009404	−0.608130
The number of surface maxima						
1	−0.03089407	−0.840670	−19.386338	−3.105421	−2.623008	−2.481294
2	−0.03090770	−0.841041	−19.394889	−3.075994	2.620312	2.503810
3	0.08174325	2.224347	51.294705	1.192296	−0.991177	−3.075460
4*	0.08360884	2.275112	52.465384	1.208705	−3.133528	−0.842281
5	0.08173091	2.224011	51.286966	1.262175	0.954538	3.048732
6	0.08359225	2.274661	52.454976	1.206454	3.133335	0.875216
7	0.04948462	1.346545	31.052094	4.464174	−1.149357	2.173051
8	0.05189294	1.412079	32.563339	6.387166	−1.413702	−1.459939
9	0.05190231	1.412334	32.569218	6.341592	1.385816	1.528097

*denotes that two extreme points are minimum and maximum, respectively.

**Table 13:** Extreme point information of the molecular surface electrostatic potential of Pt(en)Cl_4_.

The number of surface minima				Coordinate (Å)		
NO	a.u.	eV	kcal/mol	*X*	*Y*	*Z*
1	0.05905486	1.606964	37.057513	−3.668279	−1.879485	−1.503903
2	0.05909435	1.608039	37.082293	−3.658914	1.849486	1.550562
3*	−0.07459330	−2.029787	−46.808043	3.211320	−0.020578	−0.060634
The number of surface maxima						
1	0.07655763	2.083239	48.040676	−4.477896	−0.300162	2.026084
2	0.07655906	2.083278	48.041575	−4.447085	0.379344	−2.015790
3	0.08636976	2.350241	54.197890	−2.507247	−1.499431	2.173634
4*	0.08635681	2.349888	54.189761	−2.476581	1.541187	−2.161995
5	0.08720660	2.373012	54.723015	−1.594079	−1.502998	−2.994268
6*	0.08723488	2.373782	54.740760	−1.585915	1.471309	3.024505
7	−0.02777385	−0.755765	−17.428368	2.394673	−0.309417	−3.630762
8	−0.02782332	−0.757111	−17.459412	2.407678	0.231795	3.630572

*denotes that two extreme points are minimum and maximum, respectively.

**Figure 8:**
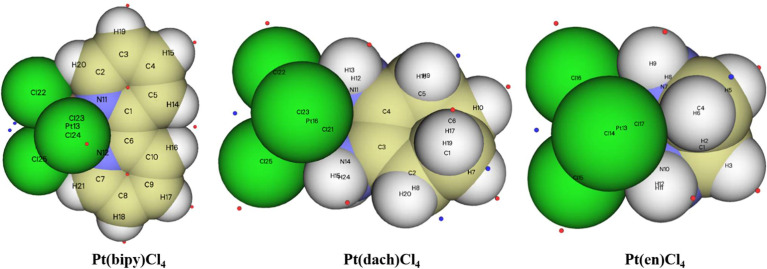
The extreme points of Pt(bipy)Cl_4_, Pt(dach)Cl_4_, and Pt(en)Cl_4_.

The extreme points around each complex are clearly shown in **Figure 8**. Red dots represent maximum points and blue dots represent minimum points. In Pt(bipy)Cl_4_, the maximum points of the surface electrostatic potential are distributed around the H atom because the electronegativity of the H atom is less than that of the C and N atoms. This is why the H atom in the system shows a positive charge. It can be seen from the position of the two minimum points and the area covered by the atomic sphere that the electrostatic potential of the molecular surface near the Pt and Cl atoms is negative. Therefore, the region of the Pt and Cl atoms is easily attacked by nucleophiles. In Pt(dach)Cl_4_ and Pt(en)Cl_4_, the region of the H atom connected with the N atom has a larger electrostatic potential and a stronger positive charge. This region leads to weak electronegativity in some parts connected with Pt and Cl atoms. The effect of these parts on the electrostatic potential is not obvious. This explains why the central Pt atom region of Pt(dach)Cl_4_ and Pt(en)Cl_4_ shows little electrophilicity. Due to the strong tension of the C-C bond, the minimum points of the surface electrostatic potential appear on the molecular surface near the C-C bond in Pt(dach)Cl_4_ and Pt(en)Cl_4_. Because the ring has a strong tension, the electron density aggregation region between C-C is “squeezed” out to some extent, not completely on the C-C bond. It is the negative contribution of this part of the electron density to the electrostatic potential that produces the minimum electrostatic potential on the molecular surface.

### Shaded Surface Map With a Projection Effect of the Electron Localization Function and the Localized Orbital Locator Analysis

The Multiwfn 3.8 tool is used to analyze the electron-depleted region and the electron-rich region by LOL projection (26, 27). Schmider and Becke first explained that the Localized Orbital Locator (LOL) is a function that is used for locating localized high localization regions (26):


$$\text{LOL}\left(\text{r}\right)=\frac{\text{τ}\left(\text{r}\right)}{1+\text{τ}\left(\text{r}\right)}$$

Where *τ*′(r) (dimensionless variable) is g0(r)/g(r). It always depends on the positive one-electron kinetic energy and is defined as


$$\text{g}\left(\text{r}\right)=\frac{1}{2}\sum {▽}_{\Psi i}\left(r\right){▽}_{\Psi i}\left(r\right)$$

where Ψi(r) is the Hartree–Fock or the Kohn Sham orbital.

**Figure 9:**
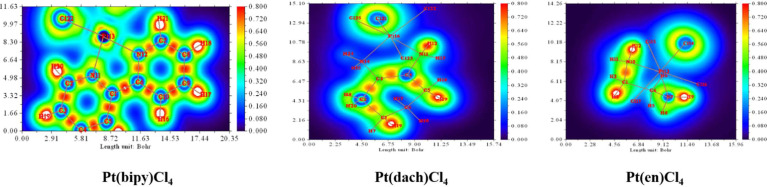
The localized orbital locator (LOL) of Pt(bipy)Cl_4_, Pt(dach)Cl_4_, and Pt(en)Cl_4_.

Sason Shaik et al. first defined the charge-shift bonds (46): When the atom binds, the kinetic energy of charge-shift bonds increases sharply and exceeds the reduction of potential energy caused by the reduction of atomic size. If atoms have lone pair electrons, kinetic energy increases more. During bonding, lone pair electrons increase kinetic energy by repulsion between lone pair electrons and bonding electrons. Therefore, the electrons in the bonding region balance the intense motion of the lone pair electrons near the atom by slower fluctuations between atoms. The LOL of Pt(bipy)Cl_4_, Pt(dach)Cl_4_, and Pt(en)Cl_4_ is plotted in **Figure 9**. The red color substantially reveals the high electron localization nature in the bonding regions. The degree of electron localization between Pt-Cl and Pt-N is not as high as the case of C-C and C-N bonds; this is a known feature of the “charge-shift bond” (47).

**Figure 10:**
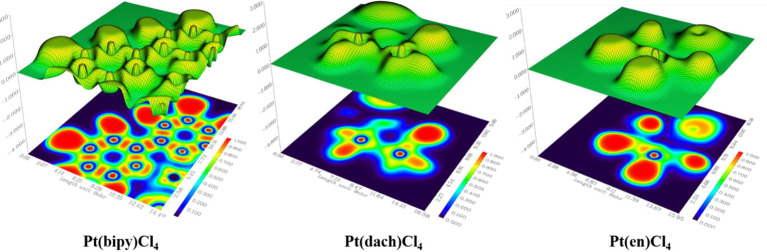
Electron localization function (ELF) of Pt(bipy)Cl_4_, Pt(dach)Cl_4_, and Pt(en)Cl_4_.

In a specific region, LOL represents the degree (strong or weak) of the limitation of the electronic movement. The shaded surface map with the projection effect of the electron localization function (ELF) of Pt(bipy)Cl_4_, Pt(dach)Cl_4_, and Pt(en)Cl_4_ is plotted in **Figure 10**. The high electron depletion region between the valence shell and the inner shell is represented by blue circles around the nucleus. Most of them appear in areas far from Pt atoms. The red-colored area represents the high localized electron regions. In order to stabilize the molecule, the electrons learn to localize on the outer side of the molecule. In **Figure 10**, we can see that electrons occupy the outside of the complexes and thus Pt(bipy)Cl_4_, Pt(dach)Cl_4_, and Pt(en)Cl_4_ are stabilized.

## Conclusion

For Pt(bipy)Cl_4_, Pt(dach)Cl_4_, and Pt(en)Cl_4_, optimized structures, theoretical frequencies, infrared intensities, HOMO- and LUMO-related physical properties, natural atomic charges, electrostatic potential extreme points, and delocalization properties are studied. The bond length of Pt-N from short to long and the bond angle of Cl-Pt-Cl from small to large are negatively correlated with the reactivity. The bond angle of Cl-Pt-N from large to small is positively correlated with the reactivity. Pt(bipy)Cl_4_ is the most easily reduced and Pt(dach)Cl_4_ is the most difficult to reduce. The order of polarizability from large to small is Pt(bipy)Cl_4_ > Pt(en)Cl_4_ > Pt(dach)Cl_4_ and softness from large to small is Pt(bipy)Cl_4_ > Pt(dach)Cl_4_ > Pt(en)Cl_4_. Pt(bipy)Cl_4_ is better than Pt(dach)Cl_4_ in terms of toxicity and dose of the drug needed to treat cancer. The degree of electron delocalization between the anti-bonding donor and the anti-bonding acceptor of Pt(bipy)Cl_4_ is higher than Pt(dach)Cl_4_ and Pt(en)Cl_4_. Then, the HOMO and LUMO orbitals of Pt(bipy)Cl_4_ have a strong delocalization. ELF and LOL analysis prove that Pt(bipy)Cl_4_, Pt(dach)Cl_4_, and Pt(en)Cl_4_ are stabilized. Our study speculates that Pt(bipy)Cl_4_ has better anticancer properties than Pt(dach)Cl_4_ and provides theoretical basis for the development of subsequent drugs.

## Data Availability Statement

The original contributions presented in the study are included in the article/supplementary material. Further inquiries can be directed to the corresponding author.

## Author Contributions

XY contributed to the conception and design of the study and wrote the first draft of the manuscript. HG supported financial technical support. All authors contributed to manuscript revision, read, and approved the submitted version.

## Funding

This work was financially supported by the Natural Science Foundation of Shandong, China (Grant No. ZR2019MC004), the High-end Talent Team Construction Foundation (Grant No. 108-10000318), and the High-end Full-time Innovative Talent Introduction Foundation “two-hundred plans” of Yantai.

## Conflict of Interest

The authors declare that the research was conducted in the absence of any commercial or financial relationships that could be construed as a potential conflict of interest.

## Publisher’s Note

All claims expressed in this article are solely those of the authors and do not necessarily represent those of their affiliated organizations, or those of the publisher, the editors and the reviewers. Any product that may be evaluated in this article, or claim that may be made by its manufacturer, is not guaranteed or endorsed by the publisher.
